# Genome-Wide Identification of U-To-C RNA Editing Events for Nuclear Genes in *Arabidopsis thaliana*

**DOI:** 10.3390/cells10030635

**Published:** 2021-03-12

**Authors:** Ruchika, Chisato Okudaira, Matomo Sakari, Toshifumi Tsukahara

**Affiliations:** 1 Area of Bioscience and Biotechnology, Japan Advanced Institute of Science and Technology, 1-1 Asahidai, Nomi City, Ishikawa 923-1292, Japan; mirandianruchika@gmail.com (R.); s1910053@jaist.ac.jp (C.O.); m-sakari@jaist.ac.jp (M.S.); 2 Division of Transdisciplinary Science, Japan Advanced Institute of Science and Technology, 1-1 Asahidai, Nomi City, Ishikawa 923-1292, Japan

**Keywords:** uridine-to-cytidine RNA editing, RNA-seq, *Arabidopsis thaliana*, differentially expressed genes (DEGs)

## Abstract

Cytosine-to-Uridine (C-to-U) RNA editing involves the deamination phenomenon, which is observed in animal nucleus and plant organelles; however, it has been considered the U-to-C is confined to the organelles of limited non-angiosperm plant species. Although previous RNA-seq-based analysis implied U-to-C RNA editing events in plant nuclear genes, it has not been broadly accepted due to inadequate confirmatory analyses. Here we examined the U-to-C RNA editing in Arabidopsis tissues at different developmental stages of growth. In this study, the high-throughput RNA sequencing (RNA-seq) of 12-day-old and 20-day-old Arabidopsis seedlings was performed, which enabled transcriptome-wide identification of RNA editing sites to analyze differentially expressed genes (DEGs) and nucleotide base conversions. The results showed that DEGs were expressed to higher levels in 12-day-old seedlings than in 20-day-old seedlings. Additionally, pentatricopeptide repeat (PPR) genes were also expressed at higher levels, as indicated by the log_2_FC values. RNA-seq analysis of 12-day- and 20-day-old Arabidopsis seedlings revealed candidates of U-to-C RNA editing events. Sanger sequencing of both DNA and cDNA for all candidate nucleotide conversions confirmed the seven U-to-C RNA editing sites. This work clearly demonstrated presence of U-to-C RNA editing for nuclear genes in Arabidopsis, which provides the basis to study the mechanism as well as the functions of the unique post-transcriptional modification.

## 1. Introduction

RNA editing, one of the most promising means of post-transcriptional gene regulation, has been widely investigated in various protozoa [1], mammalian apolipoprotein-B [2], animals [3], fungi [4], bacteria [5,6], and viruses [7,8] as well as in plants [9,10,11]. A-to-I (Inosine) RNA editing is observed in animal nuclear genes, while C-to-U RNA editing is not limited to animals but is also spreading in plant organelles. The mechanism of cytidine-to-uridine (C-to-U) RNA editing in plant organelles is completely different from that in animal nucleus but also reasonably well understood, mainly owing to the characterization of many RNA editing factors in model systems such as Arabidopsis thaliana and *Physcomitrella patens* [12]. In flowering plants, the RNA editing machinery, collectively described as the editosome, consists of at least four proteins including pentatricopeptide repeat (PPR) protein, Multiple Organellar RNA editing factor (MORF)/RNA editing factor interacting protein (RIP), Organelle RNA Recognition Motif (ORRM) proteins, and organelle zinc-finger protein (OZ1).

PPR proteins constitute a large family of proteins, with more than 400 members [13] and are either directly or indirectly responsible for RNA editing [14,15,16]. Direct selection of target sites is governed by PLS subclass PPR proteins with additional E1 and E2 domains only or further C-terminal DYW domain, which is most likely to catalyze C to U deamination.

In addition to PPR proteins, MORF/RIP, ORRM, and OZ proteins are also required for successful RNA editing and play an important role in the editosome [17]. In the *morf1* loss-of-function mutant, a single amino acid substitution in the conserved MORF domain abrogates the interaction of MORF1 with many PLS-class PPR proteins, implying that direct interaction with PPR proteins is required for the RNA editing function of MORF1 [18]. In *P. patens*, the upstream PPR stretch for RNA recognition linked in *cis* to the downstream E1, E2, and DYW domains is evident in all editing factors. Because of the simplicity of this model system, all organelle editing sites in the moss have been assigned to their corresponding DYW-type editing factors [19]. Reconstitution of target site-specific C-to-U RNA editing in *E. coli* cell as well as in vitro with a single DYW-type RNA editing factor from *Physcomitrella patens* suggests the DYW domain catalyzes the cytidine deamination.

While C-to-U RNA editing occurs in chloroplasts and mitochondria of the majority of terrestrial plants, U-to-C RNA editing is rare in land plants, except in hornworts, lycophytes, and ferns, and is, therefore, referred to as an occasional phenomenon [20]. Because of its rare occurrence only in non-model plants, negligible research has been done to explore the mechanism involved in U-to-C RNA editing. Recent finding of novel types of DYW domain, which are limited to species having U-to-C editing, implies that the domains are somehow involved in amination of uridines in plant organelles [21,22]. Therefore, this study was more centered toward the expressed PPR genes. PPR proteins are involved in RNA editing of organellar transcripts. However, their expression and functional role as the editing factors at the nuclear level further need to be uncovered.

In contrast to organellar RNA editing, RNA editing in nuclear genes of plants has not been widely accepted, though it has been suggested by few analyses based on the RNA-seq data. Recently, we also reported U-to-C and A-to-guanosine or inosine (G or I) nucleotide conversions in 12-d-old whole seedlings and leaves of 21-d-old seedlings, respectively [23,24]. However, direct comparison of DNA and cDNA sequencing from the same plant material, which is indispensable to eliminate the possibility of DNA mutations or sequencing errors, was not conducted.

Here, we examined the U-to-C RNA editing in 12-day- and 20-day-old seedlings of *Arabidopsis thaliana*, which showed distinct RNA editing status at least at a single site in previous analysis. RNA-seq data can be used for sequence differences relative to the reference genome to identify both genomic SNPs and RNA editing events. The major challenge in identifying U-to-C RNA editing sites using RNA-seq data is the discrimination of RNA editing sites from genome-encoded, single-nucleotide polymorphisms and technical artifacts caused by sequencing or read-mapping errors. We comprehensively analyzed all candidates for U-to-C RNA editing by Sanger sequencing and confirmed the presence of genuine U-to-C RNA editing events in Arabidopsis nuclear genes.

## 2. Materials and Methods

### 2.1. Plant Growth Conditions and Sample Collection

Seeds of Arabidopsis thaliana col-0 were soaked in water and incubated in the dark at 4 °C for 2–3 days. Seeds were sown in paper pots containing a 1:2:1 mixture of horticultural perlite, peat moss, and vermiculite, and covered with a plastic wrap to maintain the moisture content. The pots were placed in a U-ING Green Farm hydroponic grow box (Japan Trend shop, Osaka, Japan) in a growth room at 22 °C temperature, 45% relative humidity, and a 16-h light/8-h dark cycle. After germination, water and fertilizers were supplied twice a week. Seedlings were harvested at different days and intervals.

### 2.2. RNA Extraction and cDNA Synthesis

Total RNA was extracted from seedlings using the Qiagen Plant Mini Kit (Hilden, Germany; catalog no. 74904), according to the manufacturer’s instructions, and treated with DNase (RQ1 RNase free DNase; Promega, Madison, WI, USA) to remove traces of contaminating genomic DNA. After DNase treatment, the samples were purified by phenol-chloroform extraction, followed by ethanol precipitation. The purified RNA was quantified using a NanoDrop Spectrophotometer (Thermo Fisher Scientific, Waltham, MA, USA). Subsequently, cDNA was synthesized using reverse transcriptase (Superscript III; Invitrogen, Carlsbad, CA, USA) and a random hexamer (oligo dT) primer. The sequences of forward and reverse primers are given in Table S3.

### 2.3. Library Preparation for Transcriptome Sequencing

The mRNA from 12-d- and 20-day-old samples were enriched using oligo (dT) beads. A total amount of 3 µg RNA per sample was used as input material for the RNA sample preparations. Then, total RNA was extracted and was sent to the company, Novogene Co., Ltd., for Next Generation Sequencing analysis. Sequencing libraries were generated using NEBNext^®^ Ultra™ RNA Library Prep Kit for Illumina^®^ (NEB, Ipswich, MA, USA) following manufacturer’s recommendations and index codes were added to attribute sequences to each sample. Briefly, mRNA was purified from total RNA using poly-T oligo-attached magnetic beads. Fragmentation was carried out using divalent cations under elevated temperature in NEBNext First Strand Synthesis Reaction Buffer 5X. First strand cDNA was synthesized using random hexamer primer and M-MuLV Reverse Transcriptase (RNase H –). Second strand cDNA synthesis was subsequently performed using DNA Polymerase I and RNase H. Remaining overhangs were converted into blunt ends via exonuclease/polymerase. After adenylation of 3′ ends of DNA fragments, NEBNext Adaptor with hairpin loop structure was ligated to prepare for hybridization. In order to select cDNA fragments of preferentially 150~200 bp in length, the library fragments were purified with AMPure XP system (Beckman Coulter, Beverly, MA, USA). Then 3 µL USER Enzyme (NEB, USA) was used with size-selected, adaptor-ligated cDNA at 37 °C for 15 min followed by 5 min at 95 °C before PCR. Then PCR was performed with Phusion High-Fidelity DNA polymerase, Universal PCR primers, and Index (X) Primer. At last, PCR products were purified (AMPure XP system) and library quality was assessed on the Agilent Bioanalyzer 2100 system. The workflow for library preparation and transcriptome sequencing is shown in supporting Figure S1.

#### 2.3.1. Clustering and Sequencing

The clustering of the index-coded samples was performed on a cBot Cluster Generation System using HiSeq PE Cluster Kit cBot-HS (Illumina) according to the manufacturer’s instructions. After cluster generation, the library preparations were sequenced on an Illumina Hiseq platform and 125-bp/150-bp paired-end reads were generated.

#### 2.3.2. Data Analysis

Quality control. Raw data (raw reads) of fastq format were firstly processed through in-house perl scripts. In this step, clean data (clean reads) were obtained by removing reads containing adapter, reads containing poly- N, and low-quality reads from raw data. At the same time, Q20, Q30, and GC content from the clean data were calculated, as given in supporting Table S1. All the downstream analyses were based on the clean data with high quality.

Reads mapping to the reference genome. Reference genome (TAIR 10) and gene model annotation files were downloaded from genome website directly. Index of the reference genome was built using Bowtie v2.2.3 and paired-end clean reads were aligned to the reference genome using TopHat v2.0.12. We selected TopHat as the mapping tool, as it can generate a database of splice junctions based on the gene model annotation file and, thus, a better mapping result than other non-splice mapping tools.

Quantification of gene expression level. High-throughput sequencing (HTSeq v0.6.1, University of Heidelberg, Heidelberg, Germany) was used to count the reads’ numbers mapped to each gene. Then the FPKM of each gene was calculated based on the length of the gene and reads count mapped to this gene. FPKM, expected number of fragments per kilobase of transcript sequence per millions base pairs sequenced, considers the effect of sequencing depth and gene length for the reads’ count at the same time and is currently the most commonly used method for estimating gene expression levels [25]. HTSeq software was used to analyze FPKM, indicating the gene expression levels in this experiment, using the union mode. The resulting files presented the number of genes with different expression levels and the expression level of single genes.

Differential expression analysis (For DESeq with biological replicates). Differential expression analysis of two conditions/groups (two biological replicates per condition) was performed using the DESeq R package (1.18.0). DESeq provide statistical routines for determining differential expression in digital gene expression data using a model based on the negative binomial distribution. The resulting *p*-values were adjusted using the Benjamini and Hochberg’s approach for controlling the false discovery rate. Genes with an adjusted *p*-value < 0.05 found by DESeq were assigned as differentially expressed. (For DEGSeq without biological replicates.) Prior to differential gene expression analysis, for each sequenced library, the read counts were adjusted by edgeR program package through one scaling normalized factor. Differential expression analysis of two conditions was performed using the DEGSeq R package (1.20.0). The *p* values were adjusted using the Benjamini and Hochberg method. Corrected *p*-value of 0.005 and log_2_(Fold change) of 1 were set as the threshold for significantly differential expression.

SNP analysis. Picard-tools v1.96 and samtools v0.1.18 were used to sort, mark duplicated reads, and reorder the bam alignment results of each sample. Genome Analysis Toolkit, GATK2 (v3.2) software was used to perform SNP calling. The mapping status of reads was provided in BAM files, which were visualized using the Integrative Genomics Viewer (IGV) software.

### 2.4. Sanger Sequencing

After doing PCR with equal amounts of cDNA (100 ng) in each reaction of 20 μL volume, the PCR products were purified by 1% agarose gels and the bands were cut out and frozen. DNA was purified using the QIAquick Gel Extraction kit, and concentration was measured by Nano-Drop. Sequencing of the purified DNA was performed using the Big Dye Terminator v3.1 Cycle Sequencing Kit (Thermo Fisher Technologies, Waltham, MA, USA) using the forward and reverse primers (Table S3). The raw sequencing data were analyzed using the Sequence Scanner software version 2 (Applied Biosystems) and DNADynamo software.

## 3. Results

### 3.1. Identification and Analysis of DEGs by RNA-Seq

**Figure 1:**
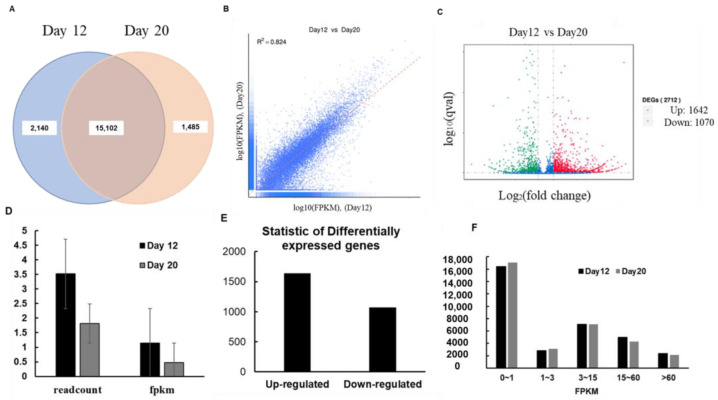
Analysis of genes differentially expressed between 12- and 20-d-old Arabidopsis seedlings. (**A**) Venn diagram of differentially expressed genes (DEGs). The sum of the numbers in each circle represents the total number of genes expressed within a sample, and the overlap represents genes expressed in both samples. (**B**) Correlation analysis of gene expression between samples. R^2^ indicates the square of the Pearson’s correlation coefficient. (**C**) Volcano plot of DEGs. The *x*-axis shows the fold change in gene expression between different samples and the *y*-axis shows the statistical significance of the differences in gene expression. Significantly up- and downregulated genes are highlighted in red and green, respectively. Genes showing no differential expression between 12- and 20-d-old seedlings are shown in blue. Comparison of the expression levels of DEGs (**D**–**F**). Comparison of, read count, and FPKM values of DEGs (**D**) between 12- and 20-d-old seedlings. (**E**) Summary of DEGs. (**F**) FPKM statistic.

The level of gene expression was measured by determining transcript abundance; the greater the transcript abundance, the higher the gene expression level [26]. In RNA-seq analysis, gene expression level is estimated by counting the number of reads mapped onto genes or exons. The lists of descriptions for all expressed genes are given in Supporting data S1. Read count was proportional not only to the actual gene expression level but also to gene length and sequencing depth. Transcriptome data indicated that a total of 33,641 genes were expressed, of which 2140 were specifically expressed in 12-day-old seedlings’ genes and 1485 in 20-day-old seedlings’ (Figure 1A). The correlation coefficient is an important indicator of the reliability of the experiment: the closer the value of the correlation coefficient to 1, the greater the similarity between samples. The square of the Pearson’s correlation coefficient (R2) should be greater than 0.92 under ideal experimental conditions. In this study, R2 was greater than 0.8, indicating a slight difference in gene expression between 12- and 20-d-old seedlings (Figure 1B). Volcano plots were used to infer the overall distribution of differentially expressed genes (DEGs). In experiments without biological replicates, the threshold is normally set as |log_2_(Fold Change)| > 1 and q-value < 0.005. By contrast, in experiments with biological replicates, DESeq eliminates biological variation; therefore, we set our threshold as adjusted *p*-value (padj) < 0.05. (Figure 1C).

The FPKM is the most well-known method of gene expression estimation in RNA-seq, as it takes into account the effects of both sequencing depth and gene length on read counts. Figure 1D shows that read counts and FPKM values were higher in 12-d-old seedlings than in the control sample (20-d-old seedlings), indicating higher expression of genes in 12-d-old seedlings. A total of 2712 genes were differentially expressed, of which 1642 were upregulated and 1070 were downregulated (Figure 1E), further indicating higher expression in 12-d-old seedlings. All DEGs are listed in Supporting data S2.

To compare gene expression levels under different conditions, an FPKM distribution diagram was used. The final FPKM value represents the mean of biological replicates. In general, an FPKM value of 0.1 or 1 was used as a threshold to determine whether a gene is expressed or not. The number of genes with different expression levels is shown in Figure 1F.

### 3.2. Comparison of Nucleotide Differences between Genomic DNA in Database and RNA-Seq of 12- or 20-D-Old Seedlings

**Figure 2:**
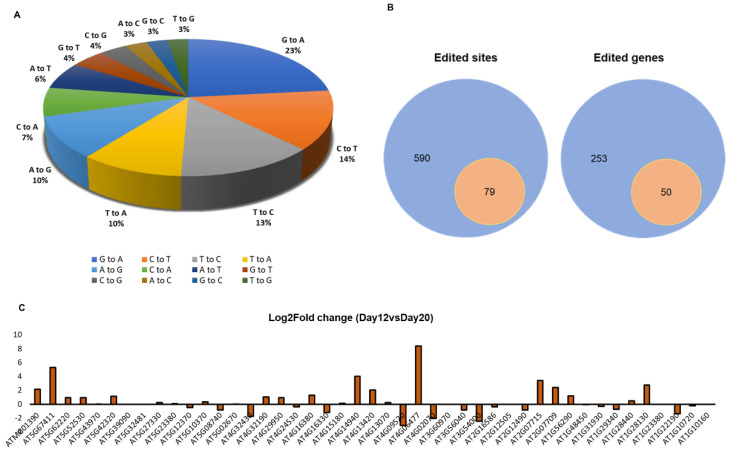
Analysis of single-nucleotide base conversions identified in 12-d-old Arabidopsis seedlings by RNA-seq. (**A**) Pie chart showing the percentage for genes identified with single-nucleotide base conversions. (**B**) Number of total edited sites and edited genes (blue), and number of sites and genes with U-to-C mutations (orange). (**C**) Log_2_FC values for the genes identified with U-to-C nucleotide conversion. Genes were expressed to higher levels in 12-d-old seedlings than in 20-d-old seedlings (**C**).

Comparison of RNA-seq data of 12-day- or 20-day-old Arabidopsis Col-0 plants to genomic DNA sequence in the database identified 12 types of possible nucleotide conversion patterns in transcripts: G-to-A, C-to-U, U-to-C, U-to-A, A-to-G, C-to-A, A-to-T, G-to-T, C-to-G, A-to-C, G-to-C, and U-to-G. Among these patterns, U-to-C was the third most predominant after G-to-A and C-to-U. Single-nucleotide base differences are listed in Supporting data S3. RNA-seq analysis revealed 590 different sites, of which 79 sites (13%) represented possible U-to-C conversion. Out of 253 genes showing nucleotide differences, 50 contained possible U-to-C conversion (Figure 2A,B). A list of candidate U-to-C RNA editing sites detected in Arabidopsis seedlings is given in Table 1.

**Figure 3:**
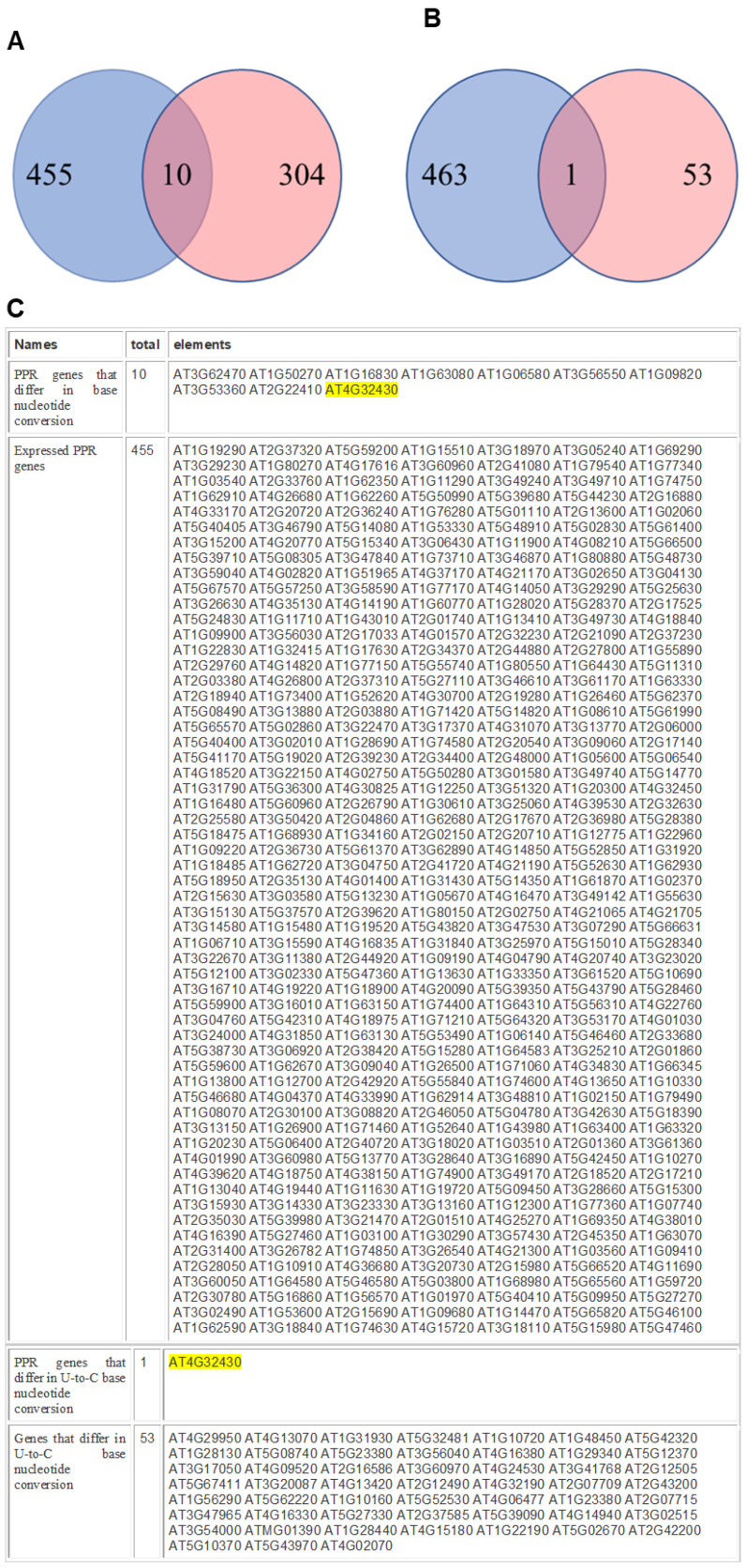
The next-generation sequencing (NGS) data of Arabidopsis for expressed *PPR* genes. Out of 465 expressed *PPR* genes, 10 genes including AT3G62470, AT1G50270, AT1G16830, AT1G63080, AT1G06580, AT3G56550, AT1G09820, AT3G53360, AT2G22410, and AT4G32430 showed nucleotide conversion (**A**). Out of 54 U-to-C variant genes, one gene, AT4G32430, was found as PPR gene (**B**). The list of expressed genes, PPR genes that differed in base nucleotide conversions, the genes that differed in U-to-C base conversion, and the PPR gene that differed in U-to-C base conversion are shown in (**C**).

Next, we analyzed the next-generation sequencing (NGS) data of Arabidopsis for expressed PPR genes using the Bioinformatics & Evolutionary Genomics website. The expressed PPR genes are listed in descending order of expression in Supporting data S4. The right column contains the genes containing all nucleotide base conversions. Out of 465 expressed PPR genes, 10 genes including AT3G62470, AT1G50270, AT1G16830, AT1G63080, AT1G06580, AT3G56550, AT1G09820, AT3G53360, AT2G22410, and AT4G32430 showed nucleotide conversion (Figure 3A). Out of 54 U-to-C variant genes, one gene, AT4G32430, was found as PPR gene (Figure 3B). The list of expressed genes, PPR genes that differed in base nucleotide conversions, the genes that differed in U-to-C base conversion, and the PPR gene that differed in U-to-C base conversion are shown in Figure 3C.

### 3.3. Identification of Genes Harboring U-To-C RNA Editing Site

**Figure 4:**
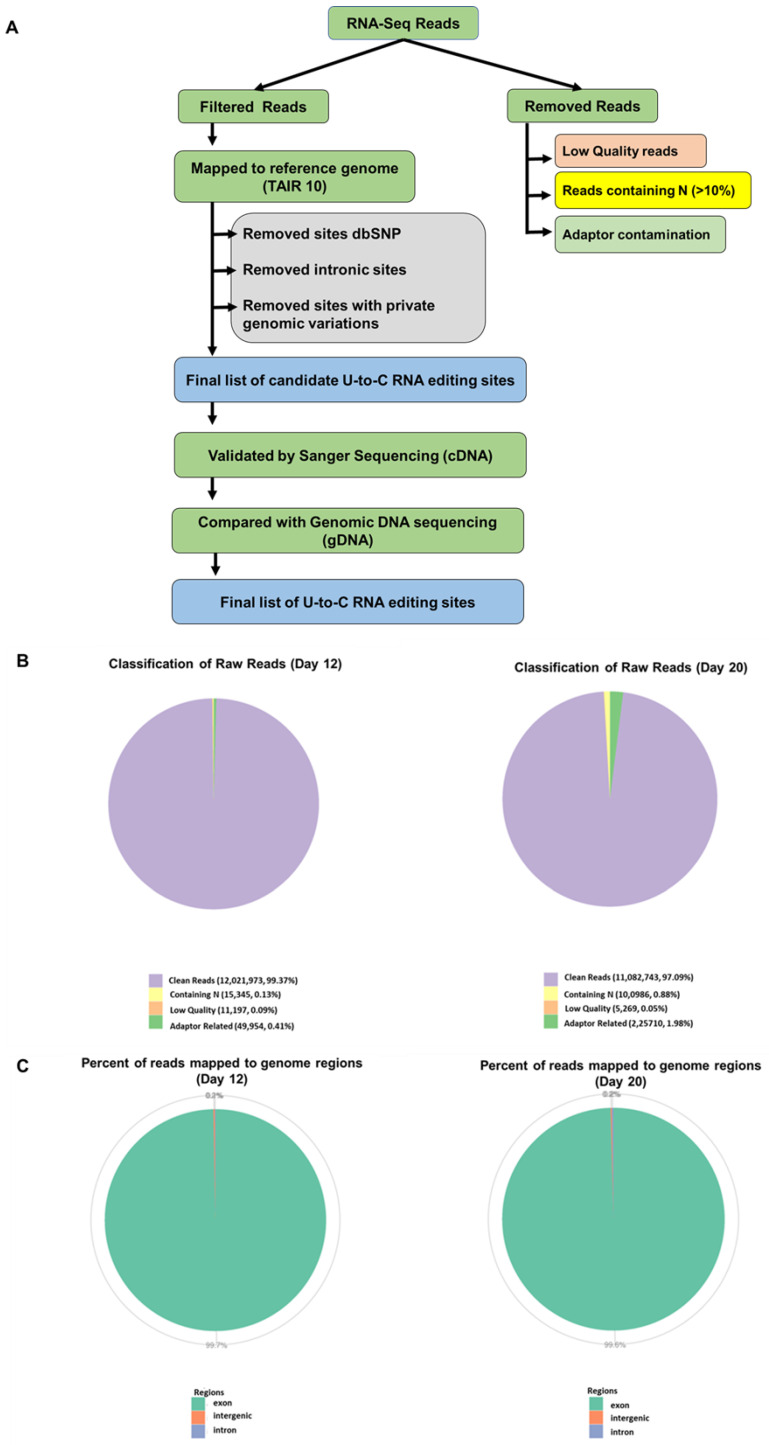
The flowchart for methodology for identification of U-to-C RNA editing site. (**A**) Raw reads are filtered to remove reads containing adapters or reads of low quality, so that downstream analyses are based on clean reads. The filtering process is as follows. (1) Discard reads with adaptor contamination. (2) Discard reads when uncertain nucleotides constitute more than 10% of either read (N > 10%). (3) Discard reads when low-quality nucleotides (base quality less than 20) constitute more than 50% of the read. For mapping sequences, TopHat2 was chosen for plant genomes. The mismatch parameter was set to 2 and other parameters were set to default. Appropriate parameters were also set, such as the longest intron length. Only filtered reads were used to analyze the mapping status of RNA-seq data to the reference genome. Edited sites were further validated and confirmed by RT-PCR. (**B**) Clean reads for day 12 and day 20. (**C**) Percentage of reads mapped to genome regions for day 12 and day 20.

**Figure 5:**
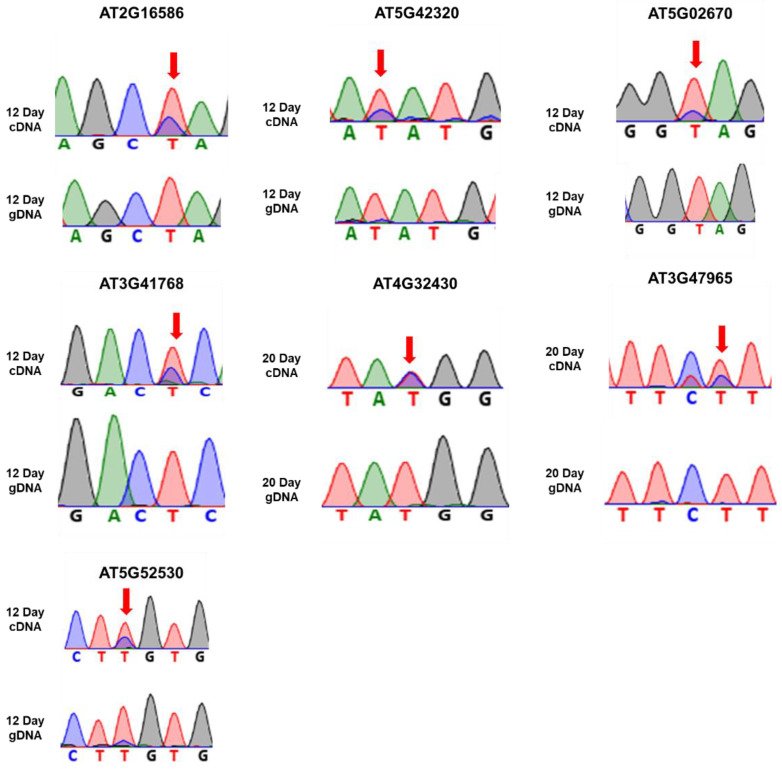
The Sanger sequence chromatogram depicting the U-to-C types of RNA editing events in 12-d- and 20-day-old seedlings from the same tissues of Arabidopsis via cDNA and genomic, gDNA using forward primers. Arrows indicate the position of RNA editing.

We selected the genes of both samples that had a minimum number of reads to be able to infer an editing event. This minimum number should be reasonably high to minimize the impact of sequencing artifacts. For example, the T-to-C change at position 14,198,871 in AT3G41768 was supported by 647(29%) and 240 (19%) in 12-d-old and 20-d-old seedlings, respectively. In addition, there were some variants that were supported by 100% of the reads in both samples (12- and 20-d-old). Therefore, these are several editing events that seem to be polymorphisms. For the same gene, we found many reads in the same U-to-C conversions. Genes with higher read coverage were further examined for the confirmation of U-to-C RNA editing sites. Genes, such as AT2G16586, AT5G02670, AT5G42320, AT4G16380, and AT5G08740, showed 249, 13, 55, 268, and 146 reads at the converted sites, respectively. Genes showing extremely low reads (0, 2) were also analyzed by RT-PCR. However, very few sites were confirmed as editing events. Because many reads mapped to each U-to-C conversion site, we considered that these nucleotide conversions were caused by RNA editing [27]. The flowchart for methodology for identification of U-to-C RNA editing sites is shown in Figure 4A. Furthermore, we validated the RNA-seq-based candidates experimentally by Sanger sequencing of both genomic, gDNA, and cDNA for all candidate genes. We extracted DNA and mRNA from the same aliquots of seedling samples. By sequencing the paired DNA and cDNA samples and analyzing each chromatogram by two individuals independently we confirmed the U-to-C RNA edited sites. The cDNA showed a double peak, representing T and edited C nucleotides, while no double peak was observed in gDNA sequencing. The sequencing was performed using sense primer targeting at the editing sites. Validation using PCR and Sanger sequencing verified seven genes, including AT2G16586, AT5G42320, AT5G02670, AT3G41768, AT4G32430, AT3G47965, and AT5G52530, containing U-to-C RNA editing sites. The Sanger sequence chromatograms for all seven edited genes are showed in Figure 5. The raw sequencing data were analyzed using the software, DNADyanamo and Sequence Scanner version 2 (Applied Biosystems). When the edited and unedited products were presented together, a dual peak (T (unedited) and C (edited)) was observed at the target site. The editing efficiency was calculated from peak area and a list of genes showing U-to-C RNA editing in 12-d- and 20-day old Arabidopsis seedlings is given in Table 2. Furthermore, we also investigated the editing efficiency at different developmental stages of Arabidopsis, such as four days and eight days. It was found that no editing occurred at early stages of development, like in four days, while a few editing could be identified in 8-day-old seedlings (Table S3). The U-to-C RNA editing sites were majorly located within the UTRs of mature mRNAs.

## 4. Discussion

In our knowledge, this is the first report of U-to-C RNA editing for nuclear genes confirmed by both RNA-seq and Sanger sequencing in flowering plants. In this study, total RNA extracted from 12-d- and 20-d-old seedlings was examined by high-throughputing RNA-seq.

Total RNA isolated from 12-d-old seedlings was examined by NGS, and DEGs were identified based on FPKM values and read counts. The results showed that DEGs were expressed to higher levels in 12-d-old seedlings than in 20-d-old seedlings. This was confirmed by higher FPKM values and read counts and more upregulated genes in 12-d-old seedlings than in 20-d-old seedlings. The ANOVA test was performed for comparing the gene expression levels. The summary for regression analysis of differentially expressed genes among the replicates of 12-d- and 20-d-old seedlings is given in Table S5. Additionally, PPR genes were also expressed to higher levels in 12-d-old than in 20-d-old seedlings, as indicated by the log_2_FC values. These data suggest that DEGs are more likely to be expressed in young Arabidopsis seedlings than in older seedlings. Therefore, more mutations could occur at this stage of development because RNA editing events are more frequent in seedlings than in any other plant tissue.

While investigating for RNA editing events to create a global map of high-quality candidates, an appropriate balance between sensitivity (identifying a highly inclusive set of possible edits) and specificity (being more confident that a call is, in fact, a true RNA edit) is required. We considered it better to have a fewer number of candidate RNA editing events that are more likely to be true than to have a larger number with an increased percentage of false positives. We undoubtedly did not score a substantial number of true, low-level, U-to-C RNA editing events in the process. Up to 90% of nucleotide variants that are not SNPs (either in dbSNP or private genomic SNVs) are U-to-C calls; this suggests they are likely to be U-to-C editing candidates. Furthermore, more than 85% of these candidates are located in UTRs. Our candidate U-to-C RNA editing sites had a different variant frequency from known SNPs. They tended to cluster predominantly in the untranslated regions.

We investigated single-nucleotide base changes and the percentage of read coverage was calculated (Table 1). We predicted 12 types of nucleotide differences, including possible U-to-C conversions. RT-PCR products of the genes including the candidate U-to-C conversions were subjected to Sanger sequencing. A total of seven genes, AT2G16586, AT5G42320, AT1G05670, AT3G41768, AT4G32430, AT3G47965, and AT5G52530, were identified as targets for U-to-C RNA editing (Table 2). The UTRs of genes encoding proteins involved in RNA metabolism and RNA binding, including PPR proteins, Zn-finger (ZnF)-related proteins, ribosomal protein L2, transmembrane proteins, and two hypothetical proteins, were identified as target of U-to-C editing. Interestingly, the ribosomal RNA, AT3G41768, was identified for 45.65% of U-to-C RNA editing efficiency. Since about 50% of genes are affected with editing, it might had had significant effect on their functions. Similarly, the transmembrane protein, AT2G16586, was identified with 77.3% of U-to-C RNA editing efficiency, which may affect its general physiology. In addition, the PPR gene, AT4G32430, was also identified with 20.43% U-to-C RNA editing.

While RNA editing in introns or UTR regions can affect mRNA stability, translation, or splicing activity because of the modification of its secondary structure, those in coding region can also affect the translated polypeptide sequence [28,29,30]. In this study, we demonstrated that most U-to-C RNA editing events are located in UTRs, which may affect the secondary structure and, consequently, the stability of mRNA.

In Arabidopsis, C-to-U and U-to-C RNA editing have been reported at the translation borders of nuclear transcripts, AT1G29930.1 and AT1G52400.1 [31]. These deamination(C-to-U) and amination (U-to-C) events accumulated at adjacent sites; therefore, the possibility that the deamination reaction serves as the amino group donor for the amination reaction was proposed, although the frequency of amination was higher than that of deamination [31]. Although this hypothesis is attractive, we could not detect the same RNA editing events in our RNA-seq data. Thus, the amino group donor of the U-to-C amination in plants is unclear. However, in cDNA AT3G47965 there is also a small T superposing with the C just upstream the edited T, showing the possible immediate donor of amino group. Previously, an extensive research on editing sites in nuclear transcripts for mRNA by Parallel Analysis of RNA Ends (PARE) and Massively Parallel Signature Sequencing (MPSS)data was reported. It showed that all 12 RNA editing patterns may exist in the nuclear genes and that perhaps the numbers of editing sites in a specific pattern may vary. The study suggested that RNA editing is an essential RNA-based regulatory layer not only for mitochondrial and chloroplast genes but also for nuclear genes. However, a global vision of RNA editing in plant nuclear protein-coding transcripts has not been realized. Therefore, this work intended to uncover the occurrence of RNA editing events in the nuclear genes of Arabidopsis.

We further compared the gene expression levels for seven identified U-to-C RNA editing target genes among different tissues (Figure S2). The green bar shows the genes expressed in seedling stage of development of Arabidopsis. The day-specific characteristic of the U-to-C RNA editing events implied that these were post-transcriptional modifications, not genomic mutations. These editings were identified as a growth-dependent RNA editing efficiency alteration. Day 4 seedlings did not have RNA editing, at least (Table S4). It indicates that the enzyme important for this editing events might have been expressed at defined stages of seedling development. Next, to validate whether the identified RNA editing sites were true positive, we searched for evidence of the identified RNA editing sites in *Arabidopsis* RNA-seq data generated by public laboratories, using online software http://signal.salk.edu/atg1001/3.0/gebrowser.php, accessed on 5 January 2021. All seven identified U-to-C RNA editing sites AT2G16586, AT5G42320, AT5G02670, AT3G41768, AT4G32430, AT3G47965, and AT5G52530 were aligned against the publicly available RNA-seq databases (Supporting data S6) and confirmed our findings. The target T sites were identified as edited C sites in various databases. The comparative analysis of *Arabidopsis* RNA-seq is shown in Figure S3. The edited sites are indicated within red boxes. Further studies are needed to better understand the processes involved in U-to-C RNA editing, including the identification of *cis* or *trans* regulatory elements, isolation of editing enzymes, and validation of editing sites.

## 5. Conclusions

Our findings confirm the uridine-to-cytidine RNA editing sites in some nuclear genes in *Arabidopsis thaliana*. A comprehensive analysis of RNA-seq data to detect nucleotide base conversions was performed. In this study, we examined U-to-C RNA editing in Arabidopsis seedlings at different developmental stages. Sanger sequencing identified the sites and efficiency of seven U-to-C editing events. Most U-to-C RNA editing here identified occurred in the UTR of mature mRNAs. Thus, we confirmed the presence of U-to-C RNA editing in nuclear genes of plants. We provided the experimental basis to explore the mechanism involved in the amination of U-to-C editing and functions and effects of U-to C RNA editing on mRNA stability, other RNA modifications, and translation.

## Figures and Tables

**Table 1:** List of candidate U-to-C RNA editing sites detected in Arabidopsis seedlings at different developmental stages.

S.No.	Position	Reads		Gene ID	Description
		12 Days	20 Days		
1	3412532	56	0	AT2G07715	Ribosomal Proteins L2, RNA binding domain
2	8544440	34	0	AT4G14940	Amine oxidase
3	26898977	2	0	AT5G67411	GRAS family transcription factor
4	8297931	4	0	AT1G23380	KNOTTED1-like homeobox gene 6
5	14657330	14	0	AT4G29950	Ypt/Rab-GAP domain of gyp1p superfamily protein
6	3392826	107	16	AT2G07709	-
7	362386	175	44	ATMG01390	-
8	7191444	105	197	AT2G16586	Unknown
9	3061212	498	2	AT4G06477	-
10	9226791	28	69	AT4G16330	2-oxoglutarate (2OG) and Fe(II)-dependent oxygenase superfamily protein
11	5816271	12	6	AT3G17050	-
12	9255546	268	99	AT4G16380	Heavy metal transport/detoxification superfamily protein
13	14198871	647	240	AT3G41768	-
14	16918673	55	46	AT5G42320	Zn-dependent exopeptidases superfamily protein
15	17708862	0	21	AT3G47965	Unknown
16	24989428	27	0	AT5G62220	glycosyltransferase 18
17	21320395	0	12	AT5G52530	dentin sialophosphoprotein-related
18	2848835	146	86	AT5G08740	NAD(P)H dehydrogenase C1
19	15546833	13	0	AT4G32190	Myosin heavy chain-related protein
20	3392918	144	14	AT2G07709	-
21	21319578	0	5	AT5G52530	dentin sialophosphoprotein-related
22	21077241	0	2	AT1G56290	CwfJ-like family protein
23	7622202	0	2	AT4G13070	RNA-binding CRS1/YhbY (CRM) domain protein
24	17692876	29	0	AT5G43970	translocase of outer membrane 22-V
25	10266697	46	6	AT1G29340	plant U-box 17
26	7869982	17	0	AT5G23380	Protein of unknown function (DUF789)
27	7836325	19	0	AT1G22190	Integrase-type DNA-binding superfamily protein
28	19998466	36	0	AT3G54000	Unknown
29	603074	13	5	AT5G02670	Unknown
30	22561577	0	2	AT3G60970	multidrug resistance-associated protein 15
31	6025041	0	27	AT4G09520	Cofactor-independent phosphoglycerate mutase
32	909133	0	2	AT4G02070	MUTS homolog 6
33	7797368	0	4	AT4G13420	high affinity K+ transporter 5
34	8662474	0	3	AT4G15180	SET domain protein 2
35	12669828	0	2	AT4G24530	O-fucosyltransferase family protein
36	15653919	0	2	AT4G32430	Pentatricopeptide repeat (PPR) superfamily protein
37	5075516	0	2	AT2G12490	-
38	17587422	0	2	AT2G42200	squamosa promoter binding protein-like 9
39	17958701	0	2	AT2G43200	S-adenosyl-L-methionine-dependent methyltransferases superfamily protein
40	526197	0	5	AT3G02515	-
41	20795012	69	64	AT3G56040	UDP-glucose pyrophosphorylase 3
42	3264804	0	2	AT5G10370	helicase domain-containing protein/IBR domain-containing protein/zinc finger protein-related
43	9633752	0	2	AT5G27330	Prefoldin chaperone subunit family protein
44	12108844	0	9	AT5G32481	-
45	15644809	0	4	AT5G39090	HXXXD-type acyl-transferase family protein
46	3332097	0	2	AT1G10160	-
47	3564739	0	2	AT1G10720	BSD domain-containing protein
48	9825469	0	6	AT1G28130	Auxin-responsive GH3 family protein
49	9997031	0	2	AT1G28440	HAESA-like 1
50	4006628	0	13	AT5G12370	exocyst complex component sec10
51	5097198	0	5	AT2G12505	-
52	11465954	0	11	AT1G31930	extra-large GTP-binding protein 3
53	7014676	0	2	AT3G20087	N/A
54	15766171	0	2	AT2G37585	Core-2/I-branching beta-1,6-N-acetylglucosaminyltransferase family protein
55	7191297	249	171	AT2G16586	Unknown
56	17908527	0	2	AT1G48450	Protein of unknown function (DUF760)

**Table 2:** List of genes identified with U-to-C RNA editing sites in 12-day- and 20-day-old Arabidopsis seedlings.

S.No.	Position	Edited Site	Gene ID	RNA Editing Efficiency (in %)		Encoded Protein
				12 Days	20 Days	
1.	14198871	5′ UTR	AT2G16586	77.30	65.74	Transmembrane protein
2.	16918673	CDS	AT5G42320	24.20	0	Zn-dependent exopeptidase superfamily protein
3.	603074	5′ UTR	AT5G02670	0	22.80	Hypothetical protein
4.	7191297	3′ UTR	AT3G41768	45.54	49.65	Ribosomal RNA
5.	15653919	3′ UTR	AT4G32430	0	20.43	PPR-like superfamily protein
6.	17708862	3′ UTR	AT3G47965	24.54	22.48	Hypothetical protein
7.	21320395	CDS	AT5G52530	20.65	0	Dentin sialophosphoprotein-like protein

## Data Availability

Not applicable.

## Supplementary Materials

The following are available online at https://www.mdpi.com/2073-4409/10/3/635/s1. Figure S1: The workflow for library preparation and transcriptome sequencing. Figure S2: Comparative analysis of gene expression levels for seven identified U-to-C RNA editing target genes among different tissues. Green bar shows the genes expressed in seedling stage of development of Arabidopsis. Figure S3: Validation of target U-to-C RNA editing sites on *Arabidopsis* RNA-seq database. Table S1: Data table for quality control. Table S2: List of candidate U-to-C RNA editing sites detected in Arabidopsis seedlings showing the percentage of read coverage. Table S3: List of candidate genes for U-to-C RNA editing sites in Arabidopsis seedlings at different developmental stages showing the forward and reverse primer sequences. Table S4: List of genes identified with U-to-C RNA editing in Arabidopsis seedlings at different developmental stages. Table S5: Summary for regression analysis of differentially expressed genes among the replicates of 12-day- and 20-day-old seedlings. Supporting data S1: Descriptions for all genes. Supporting data S2: Lists of DEGs. Supporting data S3: Lists of single-nucleotide conversions. Supporting data S4: Expressed PPR gene lists. Supporting data S5: Description for U-to-C conversion. Supporting data S6: RNA-seq database table.
