# Supplementary material for: Genome-Wide Identification of U-To-C RNA Editing Events for Nuclear Genes in Arabidopsis thaliana
Source: Cells. 2021 Mar 12;10(3):635. doi: 10.3390/cells10030635 (PMC8001311; doi:10.3390/cells10030635)
Supplement: Supplementary file 1 [file cells-10-00635-s001.zip › supplementary.v5/Supplementary files/Supporting materials_Revised.pdf]

Genome-wide identification and analysis of U-to-C RNA editing events in *Arabidopsis thaliana* by transcriptome sequencing

Ruchika[1], Chisato Okudaira[1], Matomo Sakari[1], Toshifumi Tsukahara[1,2*]

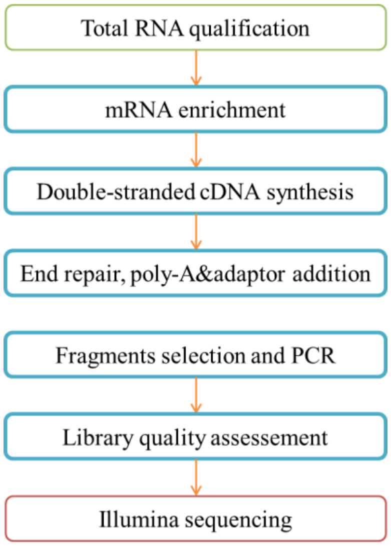

Figure S1: The workflow for library preparation and Transcriptome sequencing.

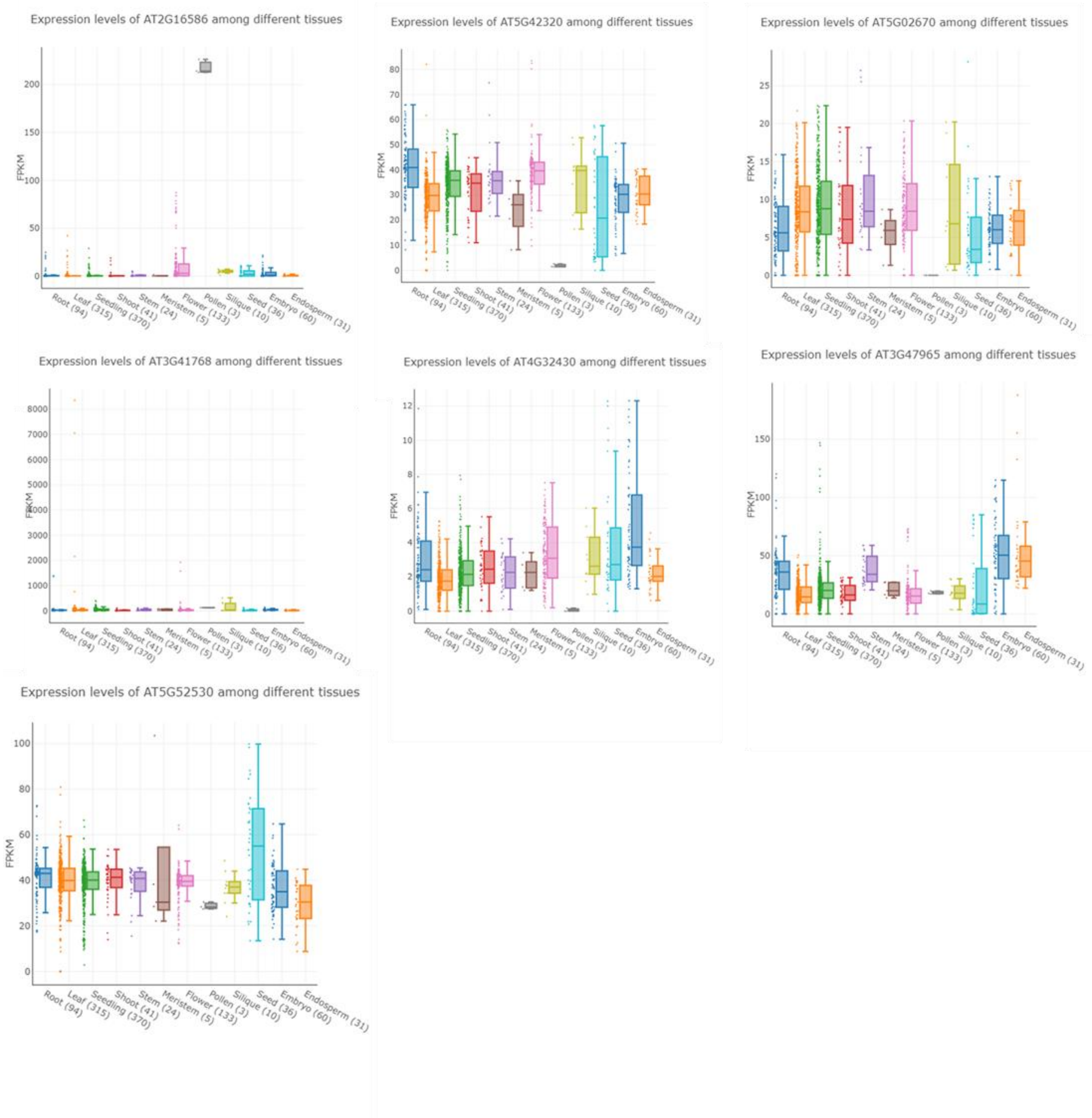

Figure S2: Comparative analysis of gene expression levels for seven identified U-to-C RNA editing target genes among different tissues. Green bar shows the genes expressed in seedling stage of development of Arabidopsis.

A) AT2G16586

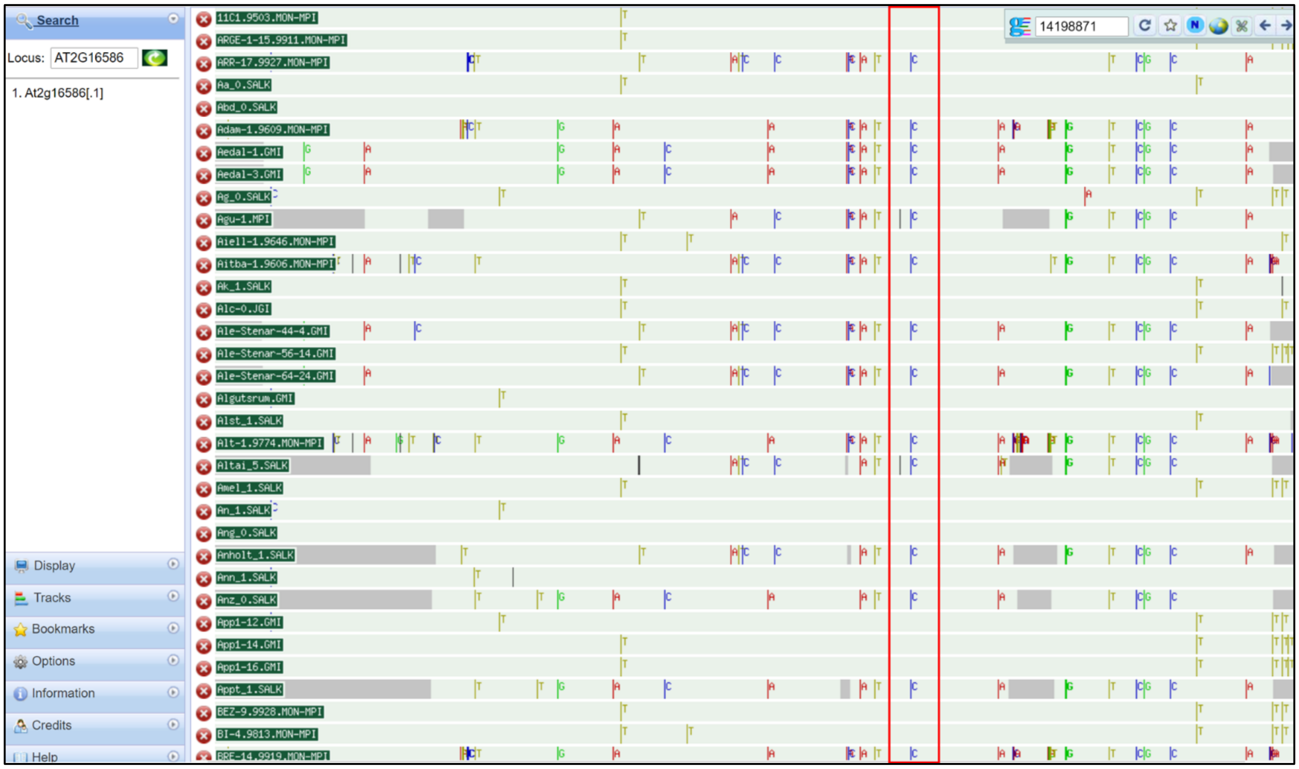

B) AT5G42320

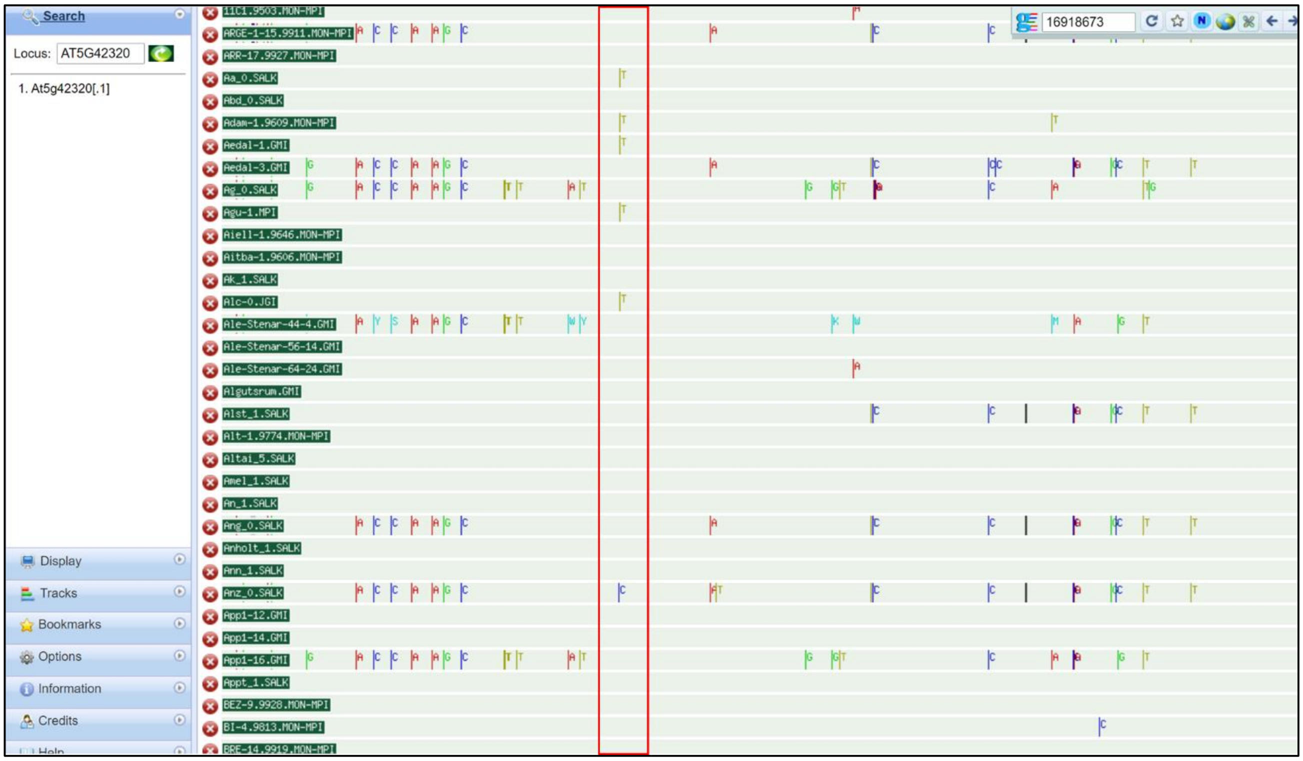

C) AT5G02670

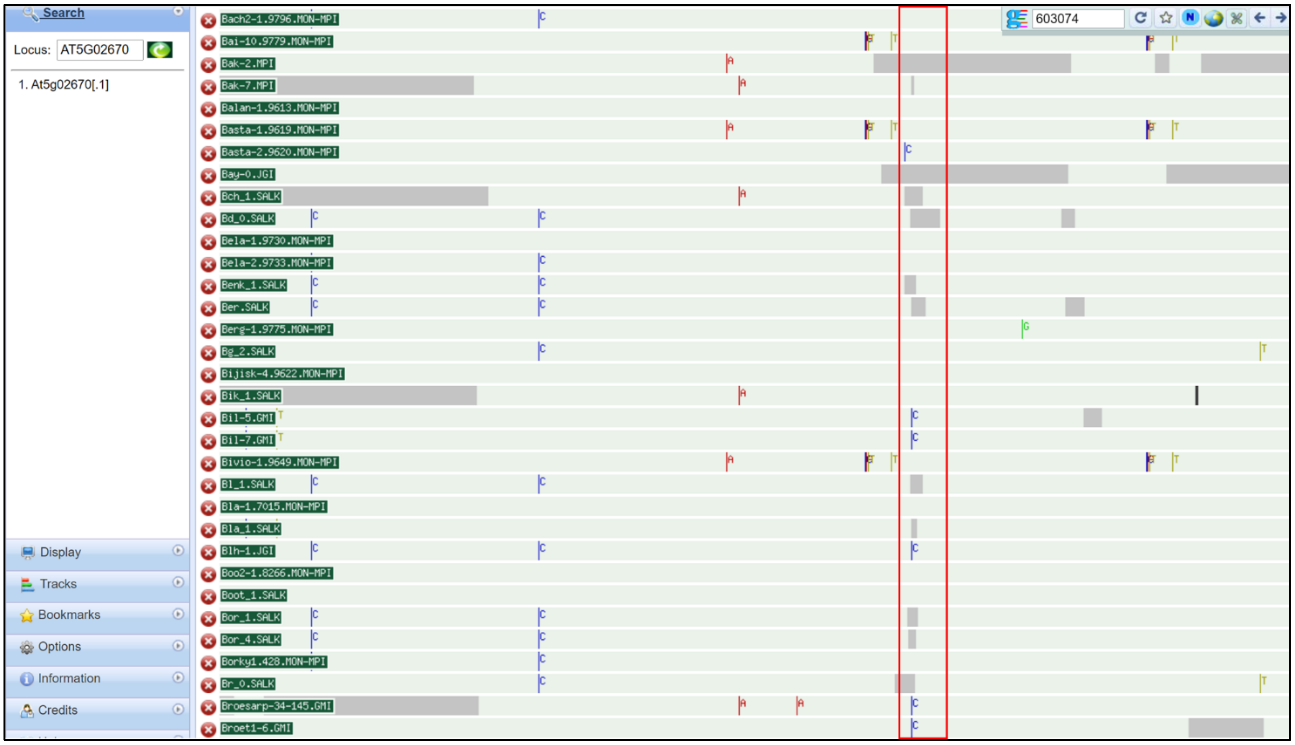

D) AT3G41768

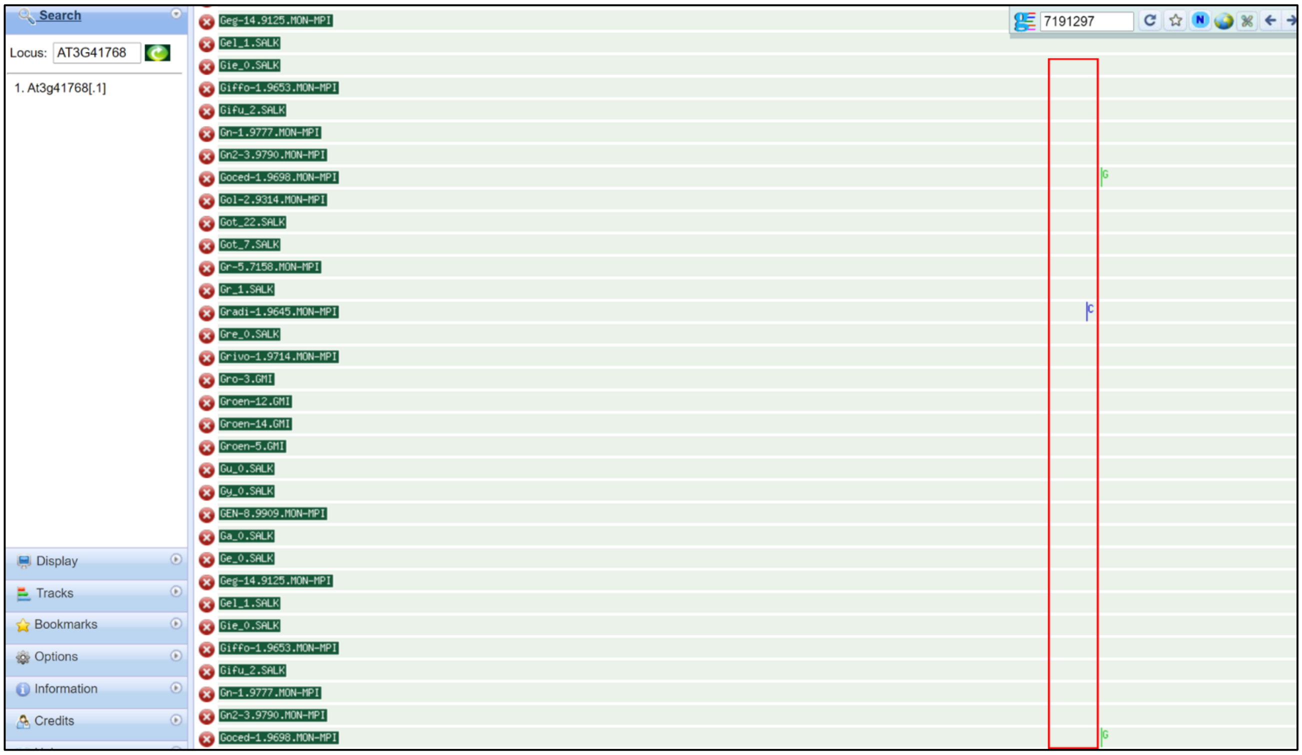

E) AT4G32430

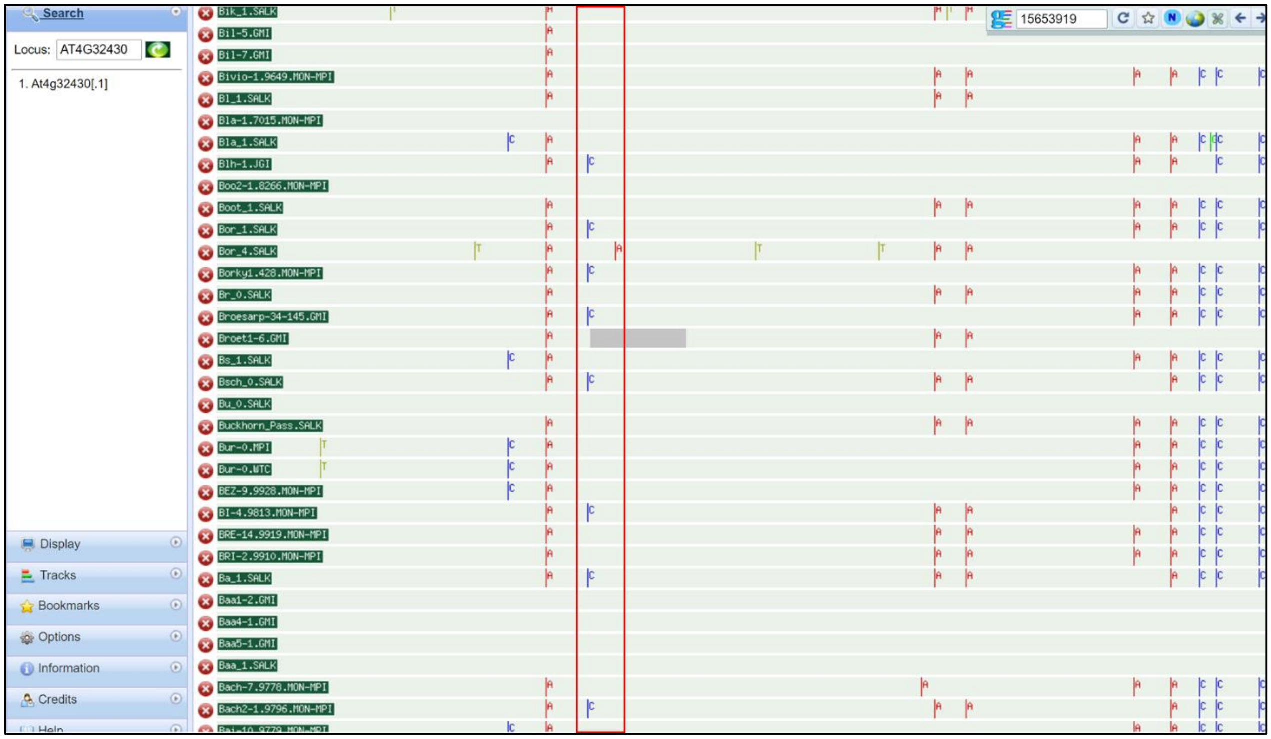

F) AT3G47965

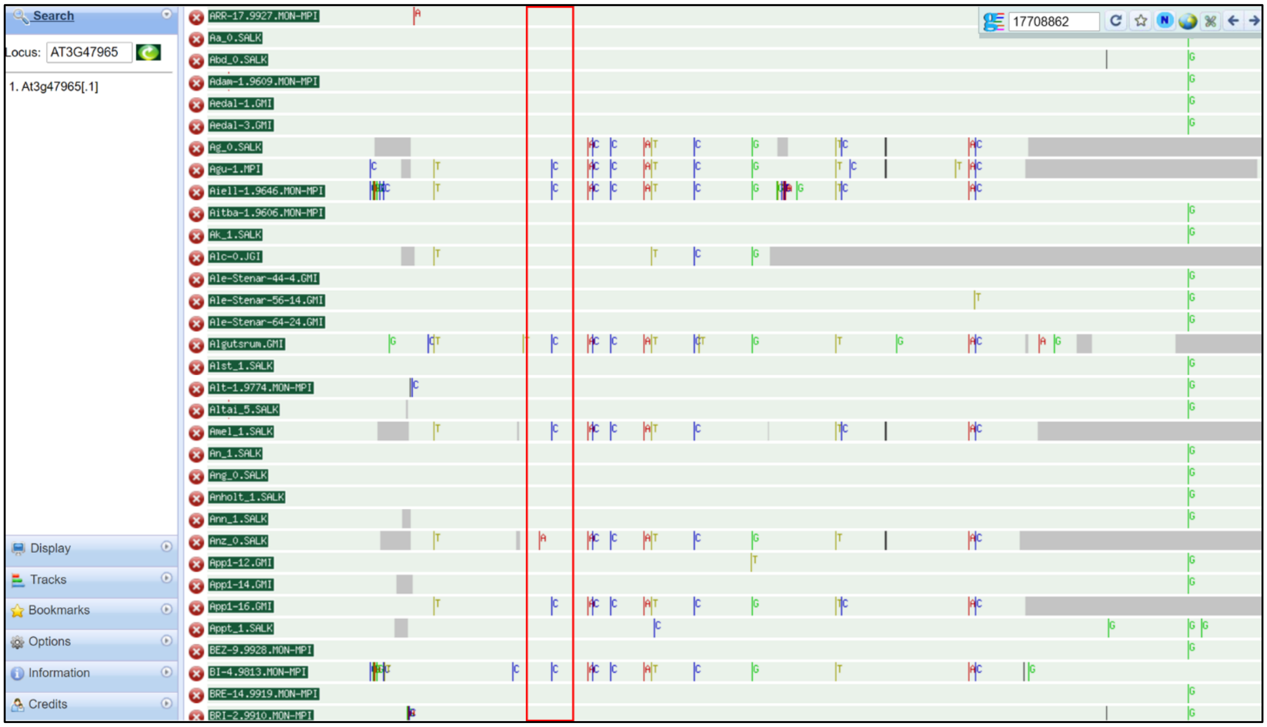

G) AT5G52530

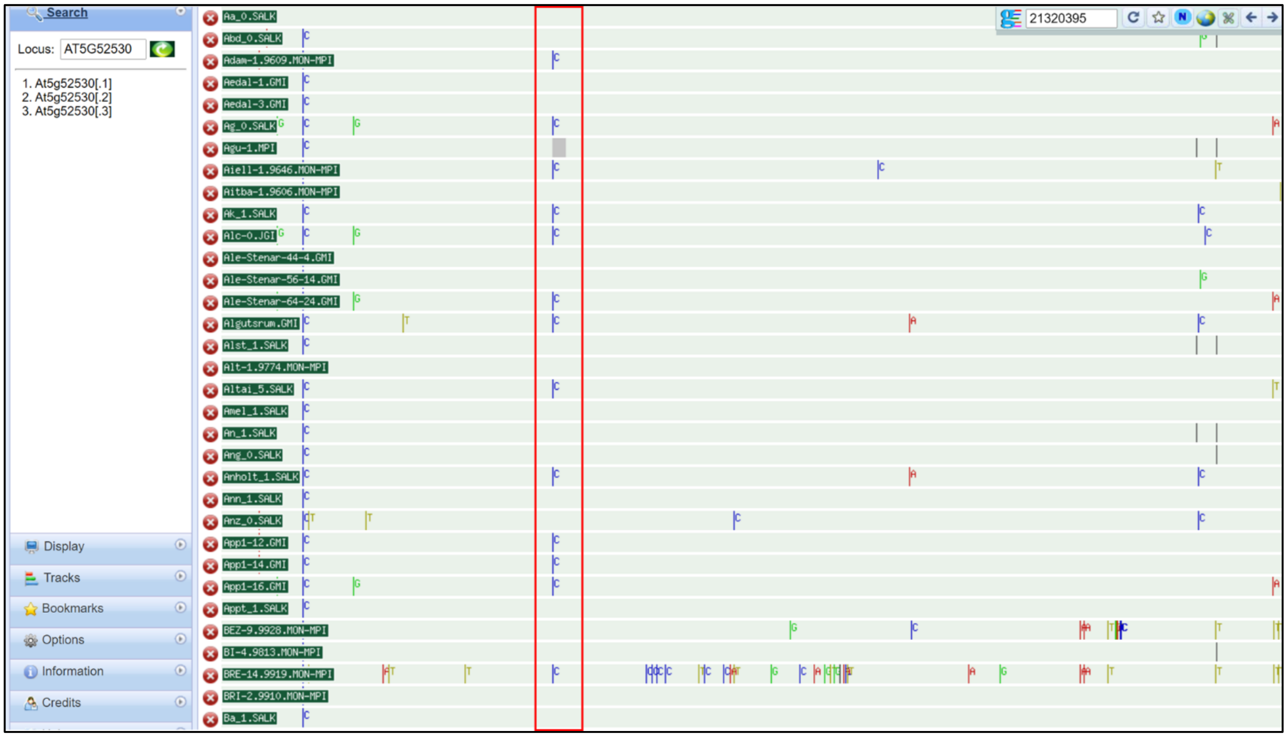

Figure S3: Validation of target U-to-C RNA editing sites on *Arabidopsis* RNA-seq database. **A)** AT2G16586, **B)** AT5G42320, **C)** AT5G02670, **D)** AT3G41768, **E)** AT4G32430, **F)** AT3G47965, **G)** AT5G5253. The identified U-to-C RNA editing sites were compared against the publicly available RNA-seq databases. The target "T" sites are identified as edited "C" sites in various databases. The edited sites are indicated within red boxes. The data is generated from an online software http://signal.salk.edu/atg1001/3.0/gebrowser.php.

Table S1: Datatable for Quality Control

Sample name	Raw reads	Clean reads	raw bases	clean bases	Error rate(%)	Q20(%)	Q30(%)	GC content(%)
Day12	24196938	24043946	3.6G	3.6G	0.03	98.03	94.06	45.88
Day20	22829416	22165486	3.4G	3.3G	0.02	98.26	94.59	45.49

Table S2. List of Candidate U-to-C RNA editing sites detected in Arabidopsis seedlings showing the percentage of read coverage.

S.No.	Accession no.	Read coverage		% Read coverage	
		12-d-old	20-d-old	12-d-old	20-d-old
1.	AT2G07715	56	0	100	0
2.	AT4G14940	34	0	100	0
3.	AT5G67411	2	0	6	0
4.	AT1G23380	4	0	33	0
5.	AT4G29950	14	0	14	0
6.	AT2G07709	107	16	100	0
7.	ATMG01390	175	44	100	0
8.	AT2G16586	105	197	99	100
9.	AT4G06477	498	2	100	100
10.	AT4G16330	28	69	100	100
11.	AT3G17050	12	6	100	100
12.	AT4G16380	268	99	100	100
13.	AT3G41768	647	240	29	19
14.	AT5G42320	55	46	36	100
15.	AT3G47965	0	21	0	19
16.	AT5G62220	27	0	37	0
17.	AT5G52530	0	12	0	55
18.	AT5G08740	146	86	99	0
19.	AT4G32190	13	0	10	100
20.	AT2G07709	144	14	100	0
21.	AT5G52530	0	5	0	0
22.	AT1G56290	0	2	0	100
23.	AT4G13070	0	2	0	26
24.	AT5G43970	29	0	32	12
25.	AT1G29340	46	6	24	13
26.	AT5G23380	17	0	100	0
27.	AT1G22190	19	0	13	2
28.	AT3G54000	36	0	73	0
29.	AT5G02670	13	5	100	31
30.	AT3G60970	0	2	0	2
31.	AT4G09520	0	27	0	31
32.	AT4G02070	0	2	0	100
33.	AT4G13420	0	4	0	2
34.	AT4G15180	0	3	0	100
35.	AT4G24530	0	2	0	100
36.	AT4G32430	0	2	0	100
37.	AT2G12490	0	2	0	0
38.	AT2G42200	0	2	0	0
39.	AT2G43200	0	2	0	50
40.	AT3G02515	0	5	0	100
41.	AT3G56040	69	64	100	100
42.	AT5G10370	0	2	0	0
43.	AT5G27330	0	2	0	0

44.	AT5G32481	0	9	0	100
45.	AT5G39090	0	4	0	0
46.	AT1G10160	0	2	0	0
47.	AT1G10720	0	2	0	0
48.	AT1G28130	0	6	0	100
49.	AT1G28440	0	2	0	100
50.	AT5G12370	0	13	0	0
51.	AT2G12505	0	5	0	0
52.	AT1G31930	0	11	0	31
53.	AT3G20087	0	2	0	100
54.	AT2G37585	0	2	0	0
55.	AT2G16586	249	171	48	100
56.	AT1G48450	0	2	0	2

Table S3. List of candidate genes for U-to-C RNA editing sites in Arabidopsis seedlings at different developmental stages.

Gene ID	Read counts		Position	Primer sequence		Length
	12days	20days		Forward	Reverse	
AT4G14940	225	186	8544440 @UTR	TTCCGGTTATGCCA ACTCTT	TGAAAGGATTTTGCCG ATGG	300bp
AT4G16380	268	99	9255546 @ CDS	ACAAACTCTGTTCC AAAGGC	GGCGGCATTCCATATCC ATT	393bp
AT4G29950	1650	713	14657330 @ UTR	GGATTCGGAGAGA GAATAAAAACG	CGGAAGAACACCGAGA TTCA	315bp
AT4G32190	1241	505	15546833 @ CDS	GAAGTCCGAACAG GCTTCTC	CTTTCCACCACCATTTC AGC	209bp
AT2G07709	107	16	3392826 @ Intron	TATTTCTTCGGAGC CGTTTC	TTCCCTTTACTAGTAGG GTG	428bp
AT2G07715	129	10	3412532 @ CDS	CCAAGCCAATAGG CGAAAGG	CGATCACTACATAAGC CGCT	310bp
AT2G16586	407	432	7191444 @ UTR	CGGTAGATTAGTTG GAACGA	GAGTAACATGGCGTTC ATGT	403bp
AT3G41768	647	240	14198871 @ UTR	CCATAAACGATGC CGACCAG	GCCTCCACGTAGCTAGT TAG	301bp
AT3G47965	91	79	17708862 @ UTR	CACATGATAGAAG CTCCTGGTG	GATACCAAAGGCGATT CAGC	153bp
AT3G54000	80	355	19998466 @ UTR	GTGGAGAGGACGA GGTTTTG	CCCGTGACAGACTGAC ATTT	122bp
AT5G02670	61	49	603074@ UTR	TTATTCACCACGAA GAAAAA	CAGGAAGCTTCCATTGT TGA	302bp
AT5G08740	146	86	2848835 @ CDS	TACTGGCTGGAATA TATGGG	GACCTTTGTGTCTCAGA GAG	380bp
AT5G23380	67	52	7869982 @ CDS	CCTCCATTTGGAGC AATGAC	GAGAGAGAGAGAGAGA TGGGAATG	237bp
AT5G43970	345	276	17692876 @ UTR	GAGGAGGCGCTCT TTATTCC	CGTCTTCCTTGGGACAC GAT	206bp

AT4G32430	07	19	156539 19@UT R	CGGTAGATTAGTTG GAACGA	GAGTAACATGGCGTTC ATGT	301bp
AT5G52530	675	293	21319578 @ CDS	TGCCAATCTGGTTA TGACTCC	GAAGGCACCTCCTTGTA TGG	291bp
AT5G52530			21320395 @ CDS	GGGATCGTAAATTC AGGGGC	CCTCACTCTTTCCGTCA TCCTC	200bp
AT5G62220	298	127	24989428 @ CDS	GCCGCTGCGTAATT TGACCA	CGCACTTTCTAACGACG GGT	197bp
AT1G22190	1169	2256	7836325 @ CDS	CCCTCCTCTCAGAA ATCTACACA	CGGAAGTACCGGTTTGT TTC	313bp
AT5G42320	420	159	16918673 @UTR	ACTCCAGTGATGAT TTAATG	TGTCTAATGTGTTTTTC AGG	302
AT1G29340	1934	2638	10266697 @ UTR	CGGATGTCTCAGTT CCGATA	AACGAACGATCACAAT GCAA	258bp
AT1G56290	190	69	21077241 @ CDS	GAAACGAAGACTC GAAGCTG	CTGGGCTTCTTCATGTT TCC	244bp
AT3G56550	20	8	20953075 @UTR	CCGGAGTCTGCAGT TATTTA	CCCGATTGAATGCTTTT GAT	231bp
AT2G16586	407	432	7191297 @ UTR	CCTAGGCTGTCCCG AAGGTA	TGTAAACCAAACTCAA CAAT	300bp

Table S4: List of genes identified with U-to-C RNA editing in Arabidopsis seedlings at different developmental stages.

S.No.	Gene ID	RNA editing efficiency (in %)			
		**4 days**	**8 days**	**12days**	**20 days**
1.	AT2G16586	0	67.54	77.30	65.74
2.	AT5G42320	0	20.47	24.20	0
3.	AT5G02670	0	0	0	22.80
4.	AT3G41768	0	40.76	45.54	49.65
5.	AT4G32430	0	0	0	20.43
6.	AT3G47965	0	0	24.54	22.48
7.	AT5G52530	0	0	20.65	0

Table S5: Summary for regression analysis of differentially expressed genes among the replicates of 12 day and 20day old seedlings.

Regression Statistics	
Multiple R	0.839863
R Square	0.705371
Adjusted R Square	0.705262
Standard Error	139.8466
Observations	2711

ANOVA

	df	SS	MS	F	Significance F
Regression	1	1.27E+08	1.27E+08	6485.604	0
Residual	2709	52980142	19557.08		
Total	2710	1.8E+08			

	Coefficients	Standard Error	t Stat	P-value	Lower 95%	Upper 95%	Lower 95.0%	Upper 95.0%
Intercept	50.41472	2.761485	18.25638	2.56E-70	44.99989	55.82955	44.99989	55.82955
5.347892	0.314265	0.003902	80.53325	0	0.306613	0.321916	0.306613	0.321916
